# Potential involvement of circulating extracellular vesicles and particles on exercise effects in malignancies

**DOI:** 10.3389/fendo.2023.1121390

**Published:** 2023-03-03

**Authors:** Ionara Rodrigues Siqueira, Rachael A. Batabyal, Robert Freishtat, Laura Reck Cechinel

**Affiliations:** ^1^ Graduate Program in Biological Sciences: Pharmacology and Therapeutics, Universidade Federal do Rio Grande do Sul, Porto Alegre, Rio Grande do Sul, Brazil; ^2^ Graduate Program in Biological Sciences: Physiology, Universidade Federal do Rio Grande do Sul, Porto Alegre, Rio Grande do Sul, Brazil; ^3^ Center for Genetic Medicine Research, Children’s National Research Institute, Washington, DC, United States; ^4^ Division of Emergency Medicine, Children’s National Hospital, Washington, DC, United States; ^5^ Department of Pediatrics, The George Washington University School of Medicine and Health Sciences, Washington, DC, United States

**Keywords:** physical activity, exosomes, microvesicles, microparticles (MP), tumor, cachexia, obesity

## Abstract

Physical activity and exercise have been widely related to prevention, treatment, and control for several non-communicable diseases. In this context, there are innumerous pre-clinical and clinical evidence indicating the potential role of exercise, beyond cancer prevention and survival, improved quality of life, including on psychological components, bone health and cachexia, from cancer survivors is described as well. This mini-review raises the potential role of circulating extracellular and particles vesicles (EVPs) cargo, as exerkines, conducting several positive effects on adjacent and/or distant tissues such as tumor, immune, bone and muscle cells. We highlighted new perspectives about microRNAs into EVPs changes induced by exercise and its benefits on malignancies, since microRNAs can be implicated with intricated physiopathological processes. Potential microRNAs into EVPs were pointed out here as players spreading beneficial effects of exercise, such as miR-150-5p, miR-124, miR-486, and miRNA-320a, which have previous findings on involvement with clinical outcomes and as well as tumor microenvironment, regulating intercellular communication and tumor growth. For example, high-intensity interval aerobic exercise program seems to increase miR‐150 contents in circulating EVPs obtained from women with normal weight or overweight. In accordance circulating EVPs miR-150-5p content is correlated with prognosis colorectal cancer, and ectopic expression of miR-150 may reduce cell proliferation, invasion and metastasis. Beyond the involvement of bioactive miRNAs into circulating EVPs and their pathways related to clinical and preclinical findings, this mini review intends to support further studies on EVPs cargo and exercise effects in oncology.

## Introduction

Exercise and/or physical activity are associated with a reduced risk of developing cancer and the improvement of well-being and survival in cancer patients (1). According to the Physical Activity Guidelines for Americans Advisory Committee, physical activity is linked to a reduced risk of several types of cancer, including breast, colon, kidney, endometrial, bladder, esophageal, and stomach cancers (2). Moreover, (3) reported that exercise reduced the risk (approximately 20%) of colorectal cancer, endometrial cancer, esophageal cancer, and kidney cancer. Preclinical studies corroborate the potential role of exercise in cancer prevention. For example, Nilsson et al. (4) reported that aged mice submitted to lifelong, voluntary wheel-running (aerobic exercise) did not exhibit malignant tumors (e.g., brain, liver, spleen, and intestinal). Cormie et al. (5) suggested a consistent trend for reduced risk of cancer-specific mortality (28%–44%), cancer recurrence (21%–35%), and all-cause mortality (25%–48%) in patients who had higher exercise levels. In addition to reducing cancer risk exercise can counteract cancer-related comorbid conditions such as cachexia, sarcopenia, osteoporosis, metabolic imbalance, and cardiovascular diseases, including those caused by cancer-related management, such as chemotherapy and radiation (5, 6).

It is noteworthy that exercise is widely accepted for the prevention and treatment of several diseases, such as cardiovascular, psychiatric, neurodegenerative, and bone, joint, and muscle disorders (7). In this context, we have focused on the role of circulating extracellular vesicles and particles (EVPs) as a potential mechanism for modulating physiological and biochemical functions in adjacent tissues and/or distant sites *via* spreading exercise-induced molecules, such as microRNAs (miRNAs). These miRNA-induced changes may promote a healthier global status (8).

Several subclasses of EVPs, such as exosomes, microvesicles, and apoptotic bodies have been described (9–11) and have the ability to transfer bioactive molecules, such as proteins, lipids, messenger RNAs (mRNAs), and miRNA (12). Recently, Sadovska et al., (13), suggested EVPs as the central mechanism of exercise induced changes in prostate cancer, specifically on the progression of cancer in a metastatic prostate cancer model. They showed that administration (i.v.) of EVPs obtained from rats submitted to regular exercise (5 weeks) reduced the tumor size (primary tumor volume by 35%) and lung metastasis using a syngeneic orthotopic prostate cancer model (13).

This mini-review will highlight the potential relationship between EVPs cargo and exercise-induced benefits on cancer conditions, given that cancer is the second-leading cause of death worldwide. We will focus on the exercise effects on EVPs cargo, bringing a new perspective on the physiological role of circulating EVPs as exerkines that spread bioactive compounds in cancer diseases.

## Role of EVPs cargo as potential mechanisms for the beneficial effects of exercise on tumor growth and metastasis

**Figure 1:**
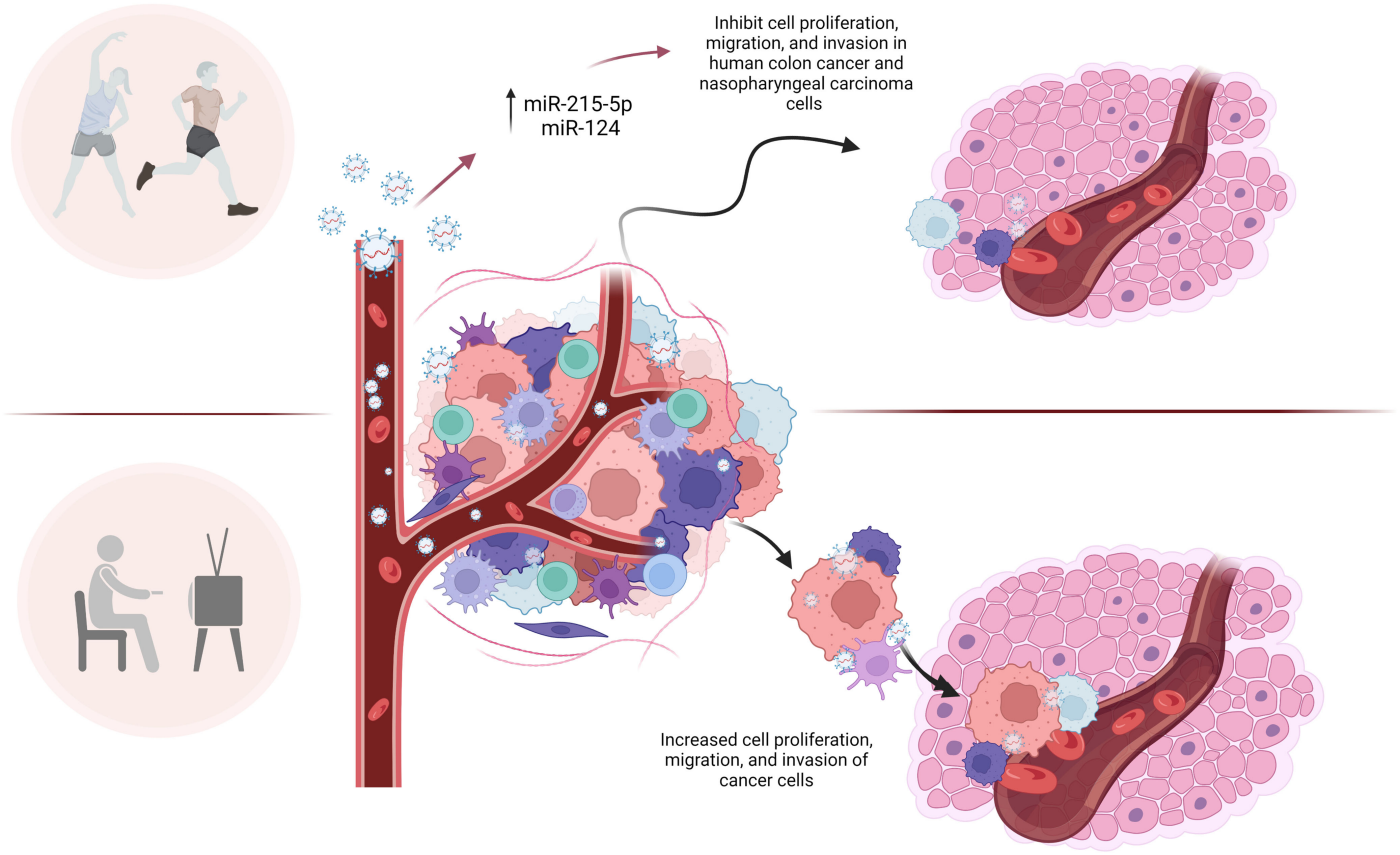
microRNAs in circulating EVPs are altered by exercise and may be candidates for the beneficial effects of exercise in tumor growth, metastasis and proliferation. microRNAs in circulating EVPs, such as miR-215-5p and miR-124, are upregulated in response to exercise in men and women, respectively. Many authors reported that those microRNAs target important molecules involved in cell growth, division, migration, and invasion of tumor, such as Yin-Yang 1 (miR-215-5p), cyclin-dependent kinase (miR-124), MMP-2 (miR-124), STAT3, p-STAT3, G1/S-specific cyclin-D2 (miR-124). Although given current data, it is impossible to affirm exactly the role of microRNAs from circulating EVPs in exercised cancer patients, it can be inferred that these molecules are involved in the effects of exercise on the tumor invasion process.

Several miRNAs in circulating EVPs impacted by exercise may be candidates for the beneficial effects of exercise in tumor growth and metastasis. Baseline levels of miR-215-5p in circulating EVPs from trained elderly men were upregulated compared to sedentary men (14). An *in vitro* study conducted by Chen et al., (15) showed that the miR-215 mimic was able to inhibit cell proliferation, migration, and invasion in human colon cancer, and these effects were reversed by its inhibitor (**Figure 1**). These authors suggested the antitumor mechanisms of miR-215 were at least in part due to reducing the expression of Yin-Yang 1 (YY1), a transcription factor belonging to the family of zinc finger proteins (15).

Another potential modulator of cell growth and division, migration, and invasion altered by exercise in circulating EVPs is miR-124 (**Figure 1**). This miRNA was upregulated in EVPs from adult women submitted to a high-intensity interval aerobic exercise program (16). The upregulation of miR-124 has also been associated with reduced proliferation, migration, and invasion of nasopharyngeal carcinoma cells (C666-1 cells) *via* downregulation of STAT3, p-STAT3, G1/S-specific cyclin-D2 (CCND2) and Matrix Metalloproteinase-2 (MMP-2) expression in C666-1 cells (17). Several molecular targets of miR-124 are key players in cell cycle progression and DNA replication, including cyclin-dependent kinase (CDK) 4 and CDK6 (18).

In addition, MMP-2, a proteinase that degrades the components of the extracellular matrix (ECM), is a target of miR-124. MMP-2 is related to the invasion of tumor cells by facilitating the movement of tumor cells across the ECM and the basal membrane of the blood vessel wall, so we can infer that MMP-2 suppression in recipient cells by higher levels of miR-124 delivered by EVPs could be involved with prevention of distant metastasis induced by exercise. MMP-2 levels in circulating blood have been considered as a breast cancer metastasis marker and are related to mortality rates (19, 20). 90% of patients that had worse prognoses (metastases and mortality), in a Brazilian cohort, had positive staining for MMP-2 in their breast cancer tumors (21), indicating they were in a “high-risk group”. Clinical and preclinical studies have demonstrated MMP-2 involvement in several human cancers, including prostate, gastric, and pancreatic cancers. In accordance, even in patients with hormone receptor-negative tumors, which are usually considered high-risk, reduced MMP-2 levels have been linked to a better prognosis (for review 22). Although given current data, it is impossible to affirm exactly the relationship between circulating miR-124 in EVPs and MMP-2 in the tumor microenvironment, it can be inferred that these molecules are involved in the effects of exercise on the tumor invasion process.

Exercise-induced miRNA changes in circulating EVPs, and repression of downstream targets, such as cyclins and CDKs mRNA, are associated with negative regulation of cell cycle progression in recipient tumor cells. Both pre-clinical and clinical evidence has demonstrated an increase in miR-486 levels in circulating EVPs from exercised mice (14, 23). Similarly, a higher level of total circulating miR-486 has been shown in resting athletes (24). Increased expression of miR-486-5p significantly decreased breast cancer cell proliferation and induced a G1 arrest, acting on its direct target the oncogene PIM-1. In addition, miR-486-5p inhibited breast cancer xenograft tumor growth in nude mice (25).

Immediately after exercise, miR-34b levels increased in circulating EVPs from trained or sedentary elderly (14). There is *in vitro* and *in vivo* evidence that miR-34-containing exosomes derived from cancer-associated fibroblasts can inhibit gastric cancer progression and development. These exosomes were taken up by gastric cancer cells, showing antitumor roles; inhibition of cell proliferation and invasion; and suppressed tumor growth *in vivo* (26). Interestingly, miR-34a acts as a tumor suppressor *via* downregulating NOX2 expression in human glioma cells showing that the miR-34a/NOX2 pathway has a relevant role in tumor proliferation (27).

Dimassi et al., (16) also described that adult Caucasian women with normal weight or overweight submitted to a high-intensity interval aerobic exercise program had increased miR‐150 levels in circulating EVPs. Decreased serum exosomal miR-150-5p expression was correlated with poor prognosis in colorectal cancer (28). In accordance, the miR-150 expression in osteosarcoma tissues was significantly decreased compared with adjacent noncancerous tissues while the expression levels of a direct target of miR-150, insulin-like growth factor 2 mRNA-binding protein 2 (IGF2BP2) were markedly increased in osteosarcoma tissues (29) and oral cancer (30). IGF2BP2, which may be controlled by miR-150, seems to be involved with maintenance of cancer stem cells, and consequently with chemoresistance and tumor recurrence (31). Jin et al., (32) described that epithelial ovarian cancer tissues and serum from patients had dramatically downregulated levels of this miRNA. The authors also found that ectopic expression of miR-150 may reduce cell proliferation, invasion, and metastasis by suppressing the expression of Zinc Finger E-Box Binding Homeobox 1 (ZEB1), a crucial regulator of epithelial-to-mesenchymal transition. Exercise-induced higher levels of miR-150 in circulating EVPs may repress IGF2BP2 and ZEB1 expression in target tissues, which may mediate the effects of exercise on some cancer types.

Moreover, miR-150 is able to improve endothelial barrier function by suppressing angiopoietin-2 (Ang2), an endothelial growth factor; miR-150 and/or Ang2 modulation have been considered promising tools for promoting improvements of endothelial barrier dysfunction in different conditions, such as sepsis and lung injury (33). Interestingly, Ang2 may be relevant to breast cancer metastases formation, and because miRNA can downregulate Ang2, this potential mechanism might be responsible for the lower breast cancer risk and the improved outcomes (i.e. decreased metastasis formation) associated with increased physical activity.

Another miRNA that may be related to decreased cell invasion and migration is miR-320a. Interestingly, this miRNA is increased in circulating EVPs after aerobic exercise (16). Xiong et al., (34) described miR-320a as a tumor suppressor in glioma by targeting aquaporin 4, a gene that is highly expressed in gliomas and is involved with glioma progression. The ability of this miRNA to suppress tumor invasion has been described in hepatocellular carcinoma cells as well, since upregulation of miR-320a inhibits c-Myc, acting on both cellular proliferation and invasion processes (35).

## Exercise may induce changes in EVP cargo that modulate the immune system in cancer

In addition to altering tumor growth and metastasis, miRNA cargo in EVPs may also be responsible for changes in the immune system in cancer. Specifically, exercise may induce changes in miRNA levels that improve the immune response to tumors. Oliveira et al., (36) showed that rats submitted to daily moderate aerobic exercise showed reduced miR-142-5p levels in circulating EVPs. Interestingly, miR-142-5p is increased in serum exosomes from patients with cervical squamous cell carcinoma compared to healthy controls (37). As discussed by Siqueira et al., (8) reduced miR-142-5p levels in EVPs can be related to counteracting the immunosuppressive tumor microenvironment condition, since this miRNA targets SOCS1 that can modulate cytokine signaling (38) and can induce a remodeling lymphatic vessel (37).

**Figure 2:**
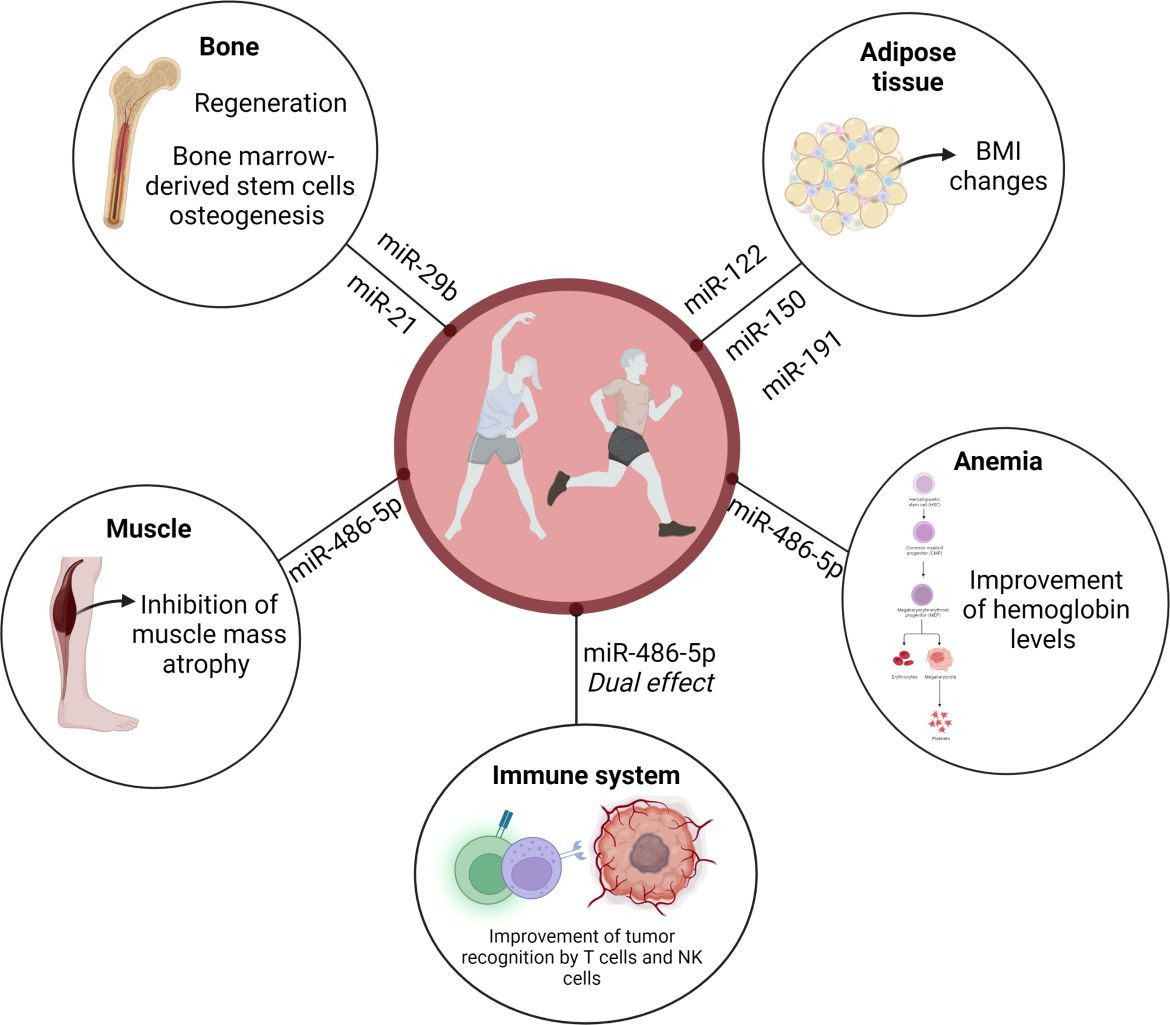
microRNA from EVP can be suggested as a possible mechanism for the beneficial effects of exercise on cancer. EVPs content may have a central role in spreading exercise-induced active molecules, such as microRNAs. miR-486 presented an exercise-induced temporal pattern, depending on inactive or trained conditions. This dual effect can be related to adaptations since this miRNA induces a relevant modulation of the immune system. miR-486-5p might be involved in improving the recognition ability of tumor cells by cytotoxic T lymphocytes and natural Killer cells in breast patients, while reduced levels induced in trained individuals briefly after exercising can avoid perpetuation of activation of immune responses. miR-486 may also regulate normal hematopoiesis. Anemia is one of the most common hematological conditions in cancer. Given the changes in this microRNA levels after exercise and the known role that miR-486-5p plays in hematopoietic progenitor growth and erythroid differentiation, we can infer that this miRNA may improve the hemoglobin levels seen in patients with cancer who exercise. As we described in this mini-review miR-29b, miR-21, miR-122, miR-150, miR-191, and miR-486-5p may be associated with changes in body composition as well as bone health in patients with cancer. Those exercise-responsive microRNAs may induce beneficial effects by improving bone healing, regeneration and promoting bone marrow-derived stem cells osteogenesis. In addition, circulating EVP miR-486 might be involved with exercise-induced muscle mass growth in cancer patients.

Findings obtained by Nair et al. (14) revealed an interesting exercise-induced temporal pattern on circulating EVP miR-486-5p levels, depending on inactive or trained conditions. The heat map reveals that the circulating EVP miR-486-5p baseline level was increased in trained elderly men compared to the sedentary control group. Although there was a reduction in trained individuals immediately after a single bout of acute exercise, the sedentary group presented a modest increase in miR-486-5p levels (14). In addition, exercise also increases miR-486 levels in circulating EVPs from exercised mice (23). This dual effect can be related to adaptations since this miRNA induces a relevant modulation of the immune system (39). In agreement, miR-486-5p has been associated with immune regulation state, for example, in the breast cancer microenvironment. miR-486-5p might be involved in improving the recognition ability of tumor cells by cytotoxic T lymphocytes and natural Killer cells in breast patients (39), while reduced levels induced in trained individuals briefly after exercising can avoid perpetuation of activation of immune responses (**Figure 2**).

## Potential role of EVPs cargo as a player in exercise-induced body composition changes in cancer

miR-486-5p shuttled by EVPs may also regulate normal hematopoiesis. It is widely accepted that anemia is one of the most common hematological conditions in cancer, induced by both cancer *per se* and myelotoxic chemotherapy, and leads to a poorer prognosis. Mohamady et al. (40) described that moderate aerobic exercise for 25–40 min, 3 times/week for 12 weeks improved hemoglobin levels and red blood cell count in elderly women with breast cancer. miR-486-5p regulates normal erythropoiesis since an important role for miR-486-5p has been described in normal hematopoietic progenitor growth and erythroid differentiation (41). Given the increase in miR-486-5p in sedentary individuals after exercise, and the baseline increase in this miRNA in active people, as well as the known role that miR-486-5p plays in hematopoietic progenitor growth and erythroid differentiation, we can infer that this miRNA may improve the hemoglobin levels seen in patients with cancer who exercise (**Figure 2**).

Another remarkable point is that overweight and obesity are associated with tumor recurrence and cancer-related mortality (42). Siqueira et al. (8) have reviewed the role of EVPs miRNAs in exercise effects on adipose tissue and consequently, obesity and overweight. Adams et al. (43) described miRNAs associated with BMI in breast cancer patients, for example, miR-191-5p, miR-150-5p, and miR-122-5p. Interestingly miR-106-5p and miR-191-5p levels were altered by a six-month weight-loss trial, including exercise. Moreover, serum miR-27a-3p was negatively correlated with total fat and total mass. However, the impact of exercise on miRNA cargo in EVPs and its association with changes in body composition in patients with cancer has not been properly explored (**Figure 2**).

Exercise-induced EVP miRNA changes may also be involved in bone health. Regular exercise has been widely highlighted and recognized as a relevant intervention to maintain or increase bone mass and density. A potential miRNA affected by exercise and involved in bone health is miR-21, since *in vitro* findings showed that miR-21 is involved with bone regeneration and promoted bone marrow-derived stem cells osteogenesis *via* the PTEN/PI3K/Akt signaling pathway (44). Circulating EVPs miR-21 is increased in individuals submitted to low-intensity exercise (45) and to a high-intensity interval aerobic exercise program (16). In addition to miR-21, higher levels of circulating EVP miR-29b were reported in sedentary elderly individuals immediately after a physical activity session (14). This miRNA has also been associated with improved bone healing in a fracture model using rodents (46) (**Figure 2**).

In addition to bone health, muscle mass atrophy (as cachexia and sarcopenia) has also been related to cancer. Morley et al. (47) suggested that approximately 20% of deaths among cancer patients are related to cachexia and its associated morbidity. Cachexia is a hypercatabolic state, defined as a multifactorial syndrome characterized by critical body loss of body weight, fat, and muscle mass related to illness (48). Sarcopenia is defined as physical performance and muscle mass loss. These terms are commonly interchanged since there are several overlaps. Interestingly, EVPs derived from bone marrow mesenchymal stem cells inhibited *in vitro* and *in vivo* dexamethasone-induced muscle atrophy through miR-486-5p/FoxO1 (49). Reduced visceral fat and increased skeletal muscle rate were described in patients with head and neck cancer after 8 weeks of multimodal exercise (50). Resistance and aerobic training have been considered relevant treatment approaches for the management of cachexia and sarcopenia (51). As described above, circulating EVPs from exercised mice had increased miR-486 levels (23), and circulating EVP miR-486-5p was increased in both trained elderly men and sedentary ones submitted to a single training session (14). It is possible to infer the involvement of circulating EVP miR-486 on exercise-induced muscle mass growth even in diseases, such as cancer (**Figure 2**).

## Factors that may alter the impact of exercised-induced EVPs mediated effects on cancer

Cancer outcomes are highly dependent on the genetic and molecular features of cancer as well as tumor cell subpopulations and staging. The literature indicates that the effects of exercise may be dependent on these factors. Jones et al. (52) described that smaller tumor sizes and lower-grade tumors can be more responsive to physical activity/exercise effects. Consequently, it is possible to infer that the impact of exercise on early tumor phases, such as inhibiting proliferation and invasion and/or improving immune response can be considered more effective in the management of cancer.

**Figure 3:**
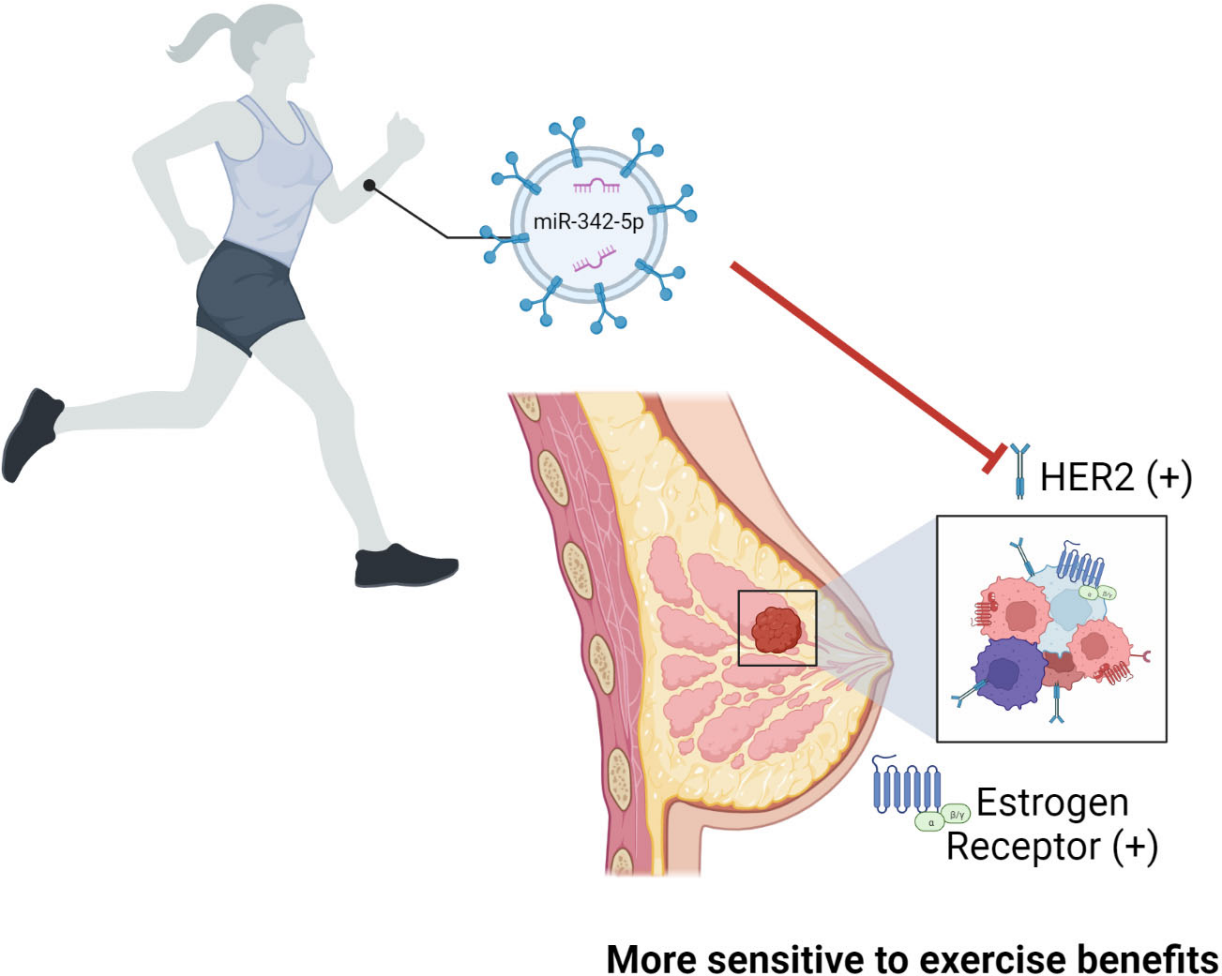
The role of miR-342-5p in breast cancer patients and its effect after exercise. Exercise was shown to increase the levels of exosomal miR-342-5p in humans submitted to 1-year rowing and rats that swam twice daily for 4 weeks. Authors reported an inhibitory effect of miR-342-5p on HER2 signaling which was associated with better overall survival and increased time to recurrence in breast cancer patients. It is relevant to point out that women diagnosed with early-stage breast cancer with estrogen receptor, progesterone receptor, or HER2 negative tumors showed reduced benefit on mortality and risk of recurrence of post-diagnosis exercise. Given that, it is possible to infer that patients with HER2-positive tumors will have higher susceptibility to physical activity/exercise benefits, although this was not tested.

In addition, miRNAs and their targets may be involved in cancer subtype-related susceptibility to physical activity/exercise benefits. Jones et al. (52) showed a lack of benefit on mortality and risk of recurrence of post-diagnosis exercise in women diagnosed with early-stage hormone or human epidermal growth factor receptor (HER2) receptor–negative breast cancer. The hazard ratio for risk of death was 0.72 and 0.50 for exercising patients with, respectively, ER and HER2-positive tumors (52). Lindholm et al. (53) demonstrated the inhibitory effects of miR-342-5p on HER2 signaling and the association between high of miR-342-5p expression and better overall survival and increased time to recurrence in breast cancer patients. To the best of our knowledge, the impact of exercise on the miRNA profile in circulating EVPs from breast cancer patients has not been tested, however, Hou et al. (54) reported that exercise increased plasma exosomal miR-342-5p in human subjects submitted to 1-year rowing training and rats that swam twice daily for 4 weeks (**Figure 3**). Given that patients with HER2-positive tumors showed increased plasma MMP2 activity, additive molecular effects of exercise such as miRNA-124 and miR-342-5p in circulating EVPs on MMP-2 levels and HER2 signaling might be explored.

## Discussion

EVP cargo can be considered as a possible mechanism for the beneficial effects of exercise on cancer. We suggest that EVPs have a central role in spreading exercise-induced active molecules, such as miRNAs, modulating healthier global condition in cancer patients. In this mini-review, we show that exercise-responsive EVP miRNAs can act synergistically to promote changes in the tumor microenvironment. The synergistic effect of these miRNAs – miR-215, miR-124, miR-486, and miR-142 – can amplify the known benefits of exercise (effects were listed on **Table 1**). In this context, miR-486 seems to have multitarget actions, such as inhibition of cell proliferation, induction of cell cycle arrest, modulation of the immune system, increase in muscle mass growth, and improvement in erythropoiesis. This mini-review intends to support further studies on EVP cargo and the effects of exercise interventions on oncologic outcomes, which may open promising avenues for further studies and perspectives on exercise in oncology.

**Table 1:** EVPs microRNAs associated with exercise and malignancie.

microRNA	Exercise findings	Proposed mechanisms of microRNA in cancer
miR-106-5p	Altered by a six-month weight-loss trial, including exercise (43)	* Associated with changes in body composition in patients with cancer (43).
miR-122-5p	Not yet explored	* Associated with BMI in breast cancer patients (43).
miR-124	Upregulated in EVPs from adult women submitted to HIIT (16).	* Reduced proliferation, migration, and invasion of nasopharyngeal carcinoma cells via STAT3, p-STAT3, CCND2, and MMP-2 (17). * CDK4 and CDK6 are miR-124 targets (18). * Impact HER2 signaling in breast cancer patients (52).
miR-142-5p	Reduced levels in rats submitted to daily moderate aerobic exercise (36).	* Increased in serum exosomes from patients with cervical squamous cell carcinoma (37). * miR-142-5p targets SOCS1, which can modulate cytokine signaling and can induce a remodeling of lymphatic vessel (37, 38).
miR-150-5p	Upregulated in EVPs from adult women submitted to HIIT with normal weight and overweight (16)	* May repress IGF2BP2 and ZEB1, which are associated with chemoresistance, tumor recurrence, cell proliferation, invasion, and metastasis (29, 30, 32)
miR-191-5p	Altered by a six-month weight-loss trial, including exercise (43).	* Associated with BMI changes in breast cancer patients (43).
miR-21	Increased after to low-intensity exercise (45) HIIT (16)	* Involved with bone regeneration and promoted bone marrow-derived stem cells osteogenesis via the PTEN/PI3K/A
miR-215-5p	Baseline levels in circulating EVPs from trained elderly men were upregulated compared to sedentary men (14).	* miR-215 mimic was able to inhibit cell proliferation, migration, and invasion in human colon cancer; antitumor mechanisms of miR-215 were at least in part due to reducing the expression of Yin-Yang 1 (15).
miR-27a-3p	Not yet explored	* Serum miR-27a-3p was negatively correlated with total fat and total mass (43).
miR-29b	Increased in sedentary elderly individuals immediately after a physical activity session (14).	* Associated with na improvement of bone healing in a fracture model using rodents (46).
miR-320a	Increased in circulating EVPs after aerobic exercise (16)	* Tumor suppressor in glioma by targeting aquaporin 4, a gene highly expressed in gliomas and involved with glioma progression (34). * Inhibits c-Myc, acting on both cellular proliferation and invasion processes (35).
miR-342-5p	Increased after 1-year rowing training and in rats that swam twice daily for 4 weeks (54).	* Upregulation inhibits HER2 and is associated with better overall survival and increased time to recurrence in breast cancer patients (53).
miR-34a/b	Increased in circulating EVPs from trained or sedentary elderly (14).	* Inhibit gastric cancer progression and development. * Have antitumor roles; inhibit cell proliferation and invasion; and suppressed tumor growth in vivo (26). * Acts as a tumor suppressor via downregulating NOX2 expression in human glioma cells (27).
miR-486-5p	* Reduction in trained individuals immediately after a single bout of acute exercise, the sedentary group presented an increase in miR-486-5p levels (14). * Increased levels in exercised mice (23).	* Decreased breast cancer cell proliferation and induced a G1 cell arrest, acting on the oncogene PIM-1. * Inhibited breast cancer xenograft tumor growth in nude mice (25). * Improved the recognition ability of tumor cells by cytotoxic T lymphocytes and natural Killer cells in breast patients, while reduced levels induced in trained individuals briefly after exercising can avoid perpetuation of activation of immune responses (39). * Regulates hematopoietic progenitor growth and erythroid differentiation (41)

## Author contributions

Conceptualization, IS, LC, RB, and RF; Writing and literature review, IS and LC; Critical revision of the manuscript, IS, LC, RB, and RF. All authors contributed to the article and approved the submitted version.
